# Machine learning in critical care: state-of-the-art and a sepsis case study

**DOI:** 10.1186/s12938-018-0569-2

**Published:** 2018-11-20

**Authors:** Alfredo Vellido, Vicent Ribas, Carles Morales, Adolfo Ruiz Sanmartín, Juan Carlos Ruiz Rodríguez

**Affiliations:** 1 Intelligent Data Science and Artificial Intelligence (IDEAI) Research Center, Universitat Politècnica de Catalunya, C. Jordi Girona, 1-3, 08034 Barcelona, Spain; 2 Centro de Investigación Biomédica en Red en Bioingeniería, Biomateriales y Nanomedicina (CIBER-BBN), Barcelona, Spain; 3 Data Analytics in Medicine, EureCat, Avinguda Diagonal, 177, 08018 Barcelona, Spain; 4 Critical Care Deparment, Vall d’Hebron University Hospital. Shock, Organ Dysfunction and Resuscitation (SODIR) Research Group, Vall d’ Hebron Research Institute (VHIR), Universitat Autònoma de Barcelona, 08035 Barcelona, Spain

**Keywords:** Critical care, Intensive care unit, Machine Learning, Sepsis

## Abstract

**Background:**

Like other scientific fields, such as cosmology, high-energy physics, or even the life sciences, medicine and healthcare face the challenge of an extremely quick transformation into data-driven sciences. This challenge entails the daunting task of extracting usable knowledge from these data using algorithmic methods. In the medical context this may for instance realized through the design of medical decision support systems for diagnosis, prognosis and patient management. The intensive care unit (ICU), and by extension the whole area of critical care, is becoming one of the most data-driven clinical environments.

**Results:**

The increasing availability of complex and heterogeneous data at the point of patient attention in critical care environments makes the development of fresh approaches to data analysis almost compulsory. Computational Intelligence (CI) and Machine Learning (ML) methods can provide such approaches and have already shown their usefulness in addressing problems in this context. The current study has a dual goal: it is first a review of the state-of-the-art on the use and application of such methods in the field of critical care. Such review is presented from the viewpoint of the different subfields of critical care, but also from the viewpoint of the different available ML and CI techniques. The second goal is presenting a collection of results that illustrate the breath of possibilities opened by ML and CI methods using a single problem, the investigation of septic shock at the ICU.

**Conclusion:**

We have presented a structured state-of-the-art that illustrates the broad-ranging ways in which ML and CI methods can make a difference in problems affecting the manifold areas of critical care. The potential of ML and CI has been illustrated in detail through an example concerning the sepsis pathology. The new definitions of sepsis and the relevance of using the systemic inflammatory response syndrome (SIRS) in its diagnosis have been considered. Conditional independence models have been used to address this problem, showing that SIRS depends on both organ dysfunction measured through the Sequential Organ Failure (SOFA) score and the ICU outcome, thus concluding that SIRS should still be considered in the study of the pathophysiology of Sepsis. Current assessment of the risk of dead at the ICU lacks specificity. ML and CI techniques are shown to improve the assessment using both indicators already in place and other clinical variables that are routinely measured. Kernel methods in particular are shown to provide the best performance balance while being amenable to representation through graphical models, which increases their interpretability and, with it, their likelihood to be accepted in medical practice.

## Background

We are witnessing an epochal change in Biology as it quickly evolves from a wet laboratory-centered science to a data-driven endeavour [1], a process that can be interpreted as an example of the pervasive Big Data paradigm [2]. Medicine draws heavily from the biological sciences and does not escape from this transformation. On the contrary, it can be argued that the transformation is enhanced by the increasing accessibility to medically-relevant data, mediated by the quick innovations on sophisticated non-invasive data acquisition technologies, and by the rapid accommodation of medical practice to advanced information technology networked systems. A further issue to consider in the context of data-centric medicine is the convergence of medicine and the omics sciences, which could assist the transition of personalized medicine from promise to reality.

Data is thus quickly becoming a main concern in medicine and its availability to medical experts is a necessary step towards the development of medical decision support systems (MDSS) [3–6]. Medical decision making in clinical environments is often made on the basis of multiple and heterogeneous parameters in the context of patient presentation. This includes the setting and the specific conditions of patient admission, as well as the medical procedures involved. Importantly, the data used in such decision making may originate from very heterogeneous sources and at multiple scales, including devices in and around the patient, medical images, laboratory information, blood tests, omics analyses, and a potential wealth of ancillary information that may be available prior to and during patient hospitalization.

A medical environments in which data dependency comes to the fore is the critical care department (CCD) in any of its general or specialized forms: intensive care unit (ICU), surgical intensive care units (SICU), neonatal intensive care unit (NICU), or pediatric intensive care unit (PICU), This has practical implications for the use of MDSS at the point of care [7]. As obvious as it may seem to say, the ICU cares for acutely ill patients. Many of these, and particularly SICU patients, are technologically dependent on life-sustaining devices such as infusion pumps, mechanical ventilators, catheters, etc. Besides treatment, the assessment of prognosis in critical care and patient stratification combining heterogeneous data sources are extremely important in an environment such as this, which is so completely centered on the patient.

The CCD conditions obviously put a strain on data acquisition and management tasks. The evaluation of clinical needs may change depending on the acuity of the patient and on the existing conditions at the point of care. The type and quantity of data captured by the bedside documentation are conditioned by changes in patient status, either through paper and electronic records, or flow sheets. It is true, though, that the medical team is ultimately responsible for the definition of what is required and, in order to support clinical decision making, it may also be necessary to include further electronic health record and monitoring devices data. These may include, amongst others, fluid intake and patient output, laboratory blood draw analyses, medical images, demographics, and so on.

Medical device connectivity in the different types of ICU is in any case necessary to provide a complete clinical decision support framework. While electronic health records offer themselves unquestionable work flow benefits, documentation and charting systems are, ultimately, only as good as the data they convey. Automated and validated data collection, achieved through seamless medical device connectivity and interoperability, supported both inside and outside the hospital premises and following the patient throughout the assisting process, should augment due diligence by care providers. In other words, it should be augmented through standardized ICU data curation.

Data analysis approaches that are tailored to the specific needs and limitations of the ICU environments are needed, and some very interesting approaches stem from the fields of Computational Intelligence (CI) and Machine Learning (ML), which have already demonstrated their capabilities both for MDSS design and development and as tools capable to improve hospital inpatient care [8].

This paper has two interrelated goals. Firstly, it aims to provide a detailed up-to-date survey on the use of CI and ML for data analysis in critical care environments. Secondly, we aim to illustrate the capabilities of these methods in the critical care domain by focusing on sepsis, a major pathology that is commonplace at the ICU.

## Methods

### Applying Machine Learning and Computational Intelligence in critical care

ML, CI and other related methodologies for data analysis, commonly described under the umbrella term of Artificial Intelligence (AI) have, over the last decades, demonstrated real value as data analysis tools in diverse biology and health-related fields. These fields include bioinformatics [9, 10], genetics and genomics [11, 12], clinical applications [13], medical decision support and clinical diagnosis [14, 15], oncology [16, 17], psychiatry and neurological disorders [18, 19], or cytopathology [20]. Note that in many of these fields, data analysis has traditionally been dispensed by statisticians and the integration of both scientific cultures has not always been a seamless process [21].

Critical care could perhaps be seen as too narrow a field to provide by itself a proper perspective on the use of ML and CI. The reality is actually very different: these types of techniques are being applied to critical care problems using approaches of remarkable depth and breadth. This situation is not new by any means: Hanson and Marshall [22] were already reviewing the use of AI in critical care back in 2001, stating that the ICU environment was particularly well suited to the deployment of AI-based analytical strategies due to the wealth of available data and the promise they hold of increased efficiency in inpatient care due to their specific characteristics.

The current paper aims to provide a state-of-the-art compact survey of ML and CI applications to problems in the area of critical care. A similar attempt by Johnson et al. [23] takes a different route and emphasizes the challenges posed by critical care data themselves. This is an interesting point of view according to which research in the field focus more on data-related challenges and arguably less on the development and application of appropriate data modelling techniques. Considering this as a Data Mining problem, we are advised to pay more attention to data understanding and pre-processing stages, before delving into the data modelling stage. Three main challenges are considered, namely *compartmentalization*, *corruption* and *complexity*. Compartmentalization includes problems related to data privacy and anonymization, data integration from potentially heterogeneous sources, and data harmonization understood as achieving a consistent definition of concepts throughout databases. Corruption would then involve different types of data errors, including data missingness and data imprecision (usually due to mismatching goals in the data acquisition and data modelling processes). Finally, complexity includes issues of state estimation, prediction, and data multi-modality, where the latter challenge draws from the stages of data pre-processing and modelling.

The current study, instead, categorizes published studies according to several different critical care subfields, singling out in sub-sections those to which more attention has been paid, and collecting the rest in a separate sub-section.

#### ML and CI for patient monitoring and alarm algorithms in critical care

As described in the introduction, critical care patients are often technologically dependent on monitoring or life-sustaining devices. The assessment of prognosis and patient stratification making use of these combined data sources is extremely important, which highlights the importance of medical device connectivity in this patient-centric environment as an essential element of a complete clinical decision support framework.

The data made available by these devices often takes the form of signal, making it the perfect target for signal processing techniques based on ML and related methods. One of the key ICU problems addressed with this approach is patient monitoring and the related design of algorithms for the implementation of patient alarms. A position editorial paper by Walsh et al. [24] recently highlighted what possibly is the single most challenging problem of this type: the potential negative effects derived from alarm fatigue; that is, from false positive alarms that might unduly mask true positive ones.

The field of alarm algorithms in critical care monitoring was considered in some detail in [25]. There, three levels at which these algorithms could operate were described, namely *signal acquisition*, *alarm generation* and *alarm validation*. Requirements for these algorithms were also listed, including: robustness against artifacts and missing values, real-time operation capabilities, predictable behavior and methodological rigor. Imhoff et al. describe their own previous early experience with ML methods in this area [26, 27]: a combination of time series analysis with support vector machines (SVM) and knowledge bases.

Artificial neural networks (ANNs) were, from very early on, picked up as building blocks of the design of monitoring alarms, specifically for monitoring of patients under anesthesia [28, 29]. A more recent study in this sub-field [30] proposed the use of Decision Trees (DT) and ANNs in the design of real-time patient-specific alarm algorithms. DTs [31] and Random Forests (RF) [32], which are an ensemble learning extension of DTs, have also been applied to the problem of reducing false cardiac arrhythmia alarms.

Other somehow less standard methods applied to these area include early work using Bayesian Networks (BN) [33] for event detection in patient monitoring. More recently, Gaussian Processes (GP) were also used for patient physiological monitoring [34] and unsupervised hierarchical clustering was applied in [35] to the identification of physiologic ICU patient states. Fuzzy systems, which could be characterized as CI methods, also under the umbrella term of AI, have been shown to be specifically well suited for the design of alarm systems at the ICU [36].

Recent work by Joshi et al. [37] in the design of alarms for the NICU is described in the next section, which is devoted to neonatal critical care. Another similar interesting line of research is that of Temko et al. concerning the NICU problem of neonatal seizure detection from EEG (see, for instance [38, 39]). In the former study, authors state that “Technologies for automated detection of neonatal seizures are gradually moving towards cot-side implementation”. For this, they propose an interesting MDSS that involves audified EEG and several ML-supported information output visualization approaches, where the output is generated through the post-processing of several EEG channel-specific SVM classifiers. Fuzzy systems for the design of NICU alarms for preterm infants were also proposed in [40].

#### Neonatal critical care

Despite the fact that we are highlighting the most popular areas of application of ML in critical care, this categorization is, to some extent, artificial because some studies cross over such categorization. An excellent example of this is the recent work by Joshi and e al. [37] who apply clustering and state-transition methods for the analysis of alarm systems in neonatal intensive care. Given that, as previously mentioned, the main problem in alarm management in critical care is the preponderance of false positive alarms, leading to “alarm fatigue”, this problem becomes specially acute in the neonatal critical care population and for premature neonates in particular due to their often far more irregular physiological signals. Another example defying easy categorization can be found in recent work by Mani et al. [41], who investigated the use of ML methods for the early detection of late-onset neonatal sepsis. This work is not just an example of crossover topics, but also of the appropriate use of a broad selection of different ML and related approach for formal comparison, including SVM, Naïve Bayes (NB) in different variants, K-Nearest Neighbor (KNN), decision trees(CART), RF, Logistic Regression (LR) and Bayesian methods.

There are certain patterns to be found in the use of ML and related methods at the NICU, both from the point of view of the specific problems addressed using these methods and from the point of view of the type of methods used. Beyond the aforementioned design and development of automated alarm systems and sepsis management, the former include problems such as risk-of-death (RoD) prediction and brain development and neurology issues in neonates, amongst others.

RoD or mortality prediction in the NICU has for instance been investigated for over a decade by Frize et al. In both [42, 43], for instance, ANNs, in their standard feed-forward multilayer perceptron (MLP) form trained by back-propagation, were used for this purpose. Interestingly, the authors define an MDSS that involves neonates’ parents in the decision loop, which is a quite unique and highly sensitive way to comply (even if inadvertently) with the new European Union General Data Protection Regulation (GDPR) that will go into effect in April 2018 [44]. The GDPR includes an article on “Automated individual decision making, including profiling” that establishes a policy on the right of citizens to receive an explanation for algorithmic decisions that may affect them.

ANNs in the latest work by Frize et al. [43] used in-built missing data imputation and treated mortality prediction as a heavily unbalanced binary classification problem (due to the comparative low mortality rate of patients in the analyzed database). Unsurprisingly in such setting, the achieved sensitivity (ratio of true positives to all positive cases) is far lower than the achieved specificity (ratio of true negatives to all negative cases). Even if surpassing clinical expectations, this reveals a typical difficulty faced by these models: if no proactive class-rebalancing procedures are used, the most relevant indicators (in this case sensitivity) are bound to under-perform.

A somehow different approach can be found in the use of ML methods for the development of MDSS for neonatal critical care assistance by Cerqueira et al. [45], where the explicit target is the prediction of the RoD for newborns admitted to NICUs, but where the emphasis is placed on the ML pipeline of the MDSS (including data preprocessing) as a simulation tool to investigate the problem beyond actual prediction. The proposed MDSS uses ANN (standard MLP) and SVM classifiers. Mortality prediction has also been addressed using DTs in [46] and Fuzzy Systems in [47].

ML has also been used for the assessment of brain development in preterm neonates at the NICU. Recent examples of this include [48, 49]. The former focuses on brain maturity prediction from functional MRI data in a dual approach that involves the use of SVM classifiers to discriminate between term- and preterm-born infants and the use of Support Vector Regression for the automated estimation of birth gestational age of neonates. The latter provides a different twist to the problem of preterm vs. term-born neonates classification, finding discriminative pattern of alterations in basal ganglia and frontal connections, first by focusing on functional connectivity patterns and, second, by assisting SVM classifiers with Independent Component Analysis (ICA)-based source extraction, in a combined source extraction-classification analytical pipeline suitable for the problem at hand. Temko et al. have a long track of research in neurological issues related to neonates at the NICU. These include for instance the recent work on neonates’ seizure detection [38, 39] from EEG that has been described in the previous section devoted to patient monitoring and alarm algorithms in critical care. In related work dealing with neonatal neurological pathologies, Ahmed, one of Temko et al. [50] investigates the non-trivial problem of grading the severity of hypoxic-ischemic encephalopathy in neonates from EEG data. Hypoxia is the lack of oxygen and ischemia the decreased blood supply to the brain near the time of birth. This severity grading analysis is understood as a multi-class problem addressed using a combination of Gaussian mixture models and SVMs.

#### ML and CI for sepsis management at the ICU

In the area of critical care, ML and other AI-based techniques have paid much attention to the problem of sepsis pathology medical management and, very specifically, to its diagnosis and prognosis, which we discuss at length in the second part of this paper.

Fuzzy systems as CI methods and rule extraction have been proposed as strategies for increasing the interpretability and usability of the results: A Fuzzy DSS for the management of post-surgical cardiac intensive care unit (CICU) patients was defined in [51]. The problem of rule generation was addressed in [52, 53], the former together with an ANN.

Beyond [52], ANNs have been applied to the study of Sepsis to produce expert systems such as the one called SES, in early work described in [54] related to the diagnosis of pathogens and prescription of antibiotics. Ross et al. [55] later proposed a system of ordinary differential equations, paired with an ANN model of inflammation and Septic Shock. Other studies have deployed ANNs for the study of sepsis. They include [56], who presented a clinical study examining SIRS and Multiple organ dysfunction syndrome (MODS) in the ICU after cardiac and thoracic surgery. A state-of-the-art application of a Deep Learning (DL) technique, namely Deep Reinforcement Learning (DRL) has recently been proposed in by Raghu et al. [57] for the definition of continuous-space models for sepsis treatment, in a twist that goes beyond the more traditional development and use of discriminative classifiers.

Another technique often used for the prediction of sepsis is the SVM. Kim et al. [58] applied this model to study the occurrence of sepsis in post-operative patients. Wang et al. [59] went a step further to build a DSS for the diagnosis of sepsis. Tang et al. [60] presented a SVM-based system for sepsis and SIRS prediction from non-invasive cardiovascular spectrum analysis.

ML methods have achieved success in varying degrees in the problem of the prediction of mortality caused by Sepsis. In [61], a Septic Shock diagnostic system based on ANNs (Radial Basis Functions—RBF- and supervised Growing Neural Gas) was introduced . Brause et al. [62], also in this area, used an evolutionary algorithm in an RBF network (the MEDAN Project) to obtain a set of predictive attributes for assessing mortality for Abdominal Sepsis over a retrospective dataset. Relevance Vector Machines (RVM), which are SVM variants with embedded feature relevance determination, were used in [63], DTs were used in [64], while BN models were used in [65, 66]. Finally, kernel methods were used in [67].

#### Further applications of ML and CI in critical care

The previous topic-specific sub-sections are devoted to those critical care-related problems to which, arguably, a closest attention has been paid in ML and CI. All these reviews reflect not only the manifold areas of application to critical care, but also the broad palette of methods available to practitioners in critical care.

Other problems, to which perhaps less attention has been paid from this point of view include, for instance, general mortality prediction using ANN models [68–70] and SVMs [71]. Another problem is that of medicine dosing, to which less standard methods such as BN [72], or DRL [73] have been used. Other applications of Deep Learning techniques (the current *reincarnation* of ANNs, very much in vogue but which is usually restricted by large data requirements) include that for the unsupervised learning of phenotypical features in longitudinal sequences of serum uric acid measurements, as investigated in [74]. The application of Fuzzy Systems for disease-based modeling for fluid resuscitation and vasopressor use in ICUs has also been investigated in [75].

## Results and discussion

### Machine Learning for the analysis of sepsis as a paradigmatic critical care pathology

Sepsis data analysis using ML and related methods has been the focus of the authors’ research in critical care. Therefore, the second part of this paper pays detailed attention to our work on the application of these methods to the solution of diverse problems in the management of sepsis. We use this work as an illustrative example of their capabilities and limitations as applied in the field.

#### Sepsis: some basic background

The official consensus definition of the sepsis pathology has substantially evolved over the last few decades. The last consensus meeting held in 2016 agreed on new definitions both for sepsis and its complications [76]. These new definitions include organ dysfunction for the diagnosis in order to increase the specificity in diagnosing sepsis in clinical practice. Consequently, the concept of severe sepsis is discontinued in clinical practice and the use of SIRS [77] for diagnosing sepsis is also not any longer recommended. In contrast, the SOFA [78] score acquires a more prominent role in the diagnosis and general management of sepsis.

Unfortunately, the calculation of the SOFA score requires to perform blood tests to assess coagulation, liver and renal function and can, as a result, be more time consuming than what the ICU environment actually affords. The quick SOFA (qSOFA) score was defined as an alternative that allows making executive decisions and admit patients into the ICU in a realistic fashion. It just assesses mental status, systolic blood pressure (SBP) and respiratory rate [76].

Sepsis is thus defined as life-threatening organ dysfunction caused by a dysregulated host response to infection and organ dysfunction can be identified as an acute change in total SOFA score $\ge 2$ points consequent to the infection. Likewise, Septic Shock is defined as a subset of sepsis in which underlying circulatory and cellular/metabolic abnormalities are profound enough to substantially increase mortality [76].

Note that these current definitions are yet to be widely adopted in day-to-day critical care. Despite this, they set the basis to validate the common ground in the study of the pathophysiology of sepsis and its prognosis at multiple levels of analysis, including the study of the inflammatory response during sepsis and its association with organ dysfunction, both from already available clinical data and at the level of transcriptomics, proteomics and metabolomics. All of this in order to achieve a better understanding the underlying mechanisms of the pathological process.

#### Reviewing the definitions of sepsis using causal probabilistic networks

A study aiming to identify key prognostic factors in patients that suffered a septic shock during their stay in the ICU is presented in this section. All analyses are based on the MEDAN database [79], which recorded data from 71 voluntary cooperating German ICUs between 1998 and 2002.

This database is publicly available and its data was analyzed using causal probabilistic networks (CPN). CPNs are accepted in computational biology and bioinformatics as capable of modeling causal relationships in a more precise fashion than standard clustering or regression models. These models also have sound statistical foundations for inferential modeling [80] that help data pre-processing tasks such as noise and missing data handling.

The practical implementation of the CPNs used the *Causal Explorer* public library [81] with the three-phase dependency analysis algorithm (TPDA). This algorithm involves three phases, namely drafting, thickening and thinning. In the drafting phase, TPDA produces an initial set of edges based on a simple test (just showing sufficient pairwise mutual information). This first draft is a graph without loops. In the second phase, TPDA adds edges to the current graph whenever the pairs of nodes under analysis cannot be separated using a set of conditional independence tests. The graph resulting from this phase contains all the edges of the underlying dependency model. Finally, in the thinning phase, each edge is examined and removed if the two nodes of the edge are found to be conditionally independent. The threshold value for our TDPA implementation was set to a 0.05 value.

**Fig. 1:**
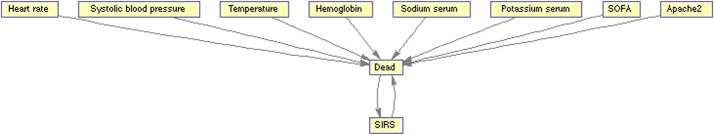
Conditional Independence Map. Conditional Independence Map for data at ICU admission

The graph displayed in Fig. 1 was obtained for ICU admission. In this graph, the dependence relations show the link between SIRS and the ICU outcome variable, hinting a strong relationship between SIRS diagnosis and patient’s ICU outcome.

**Fig. 2:**
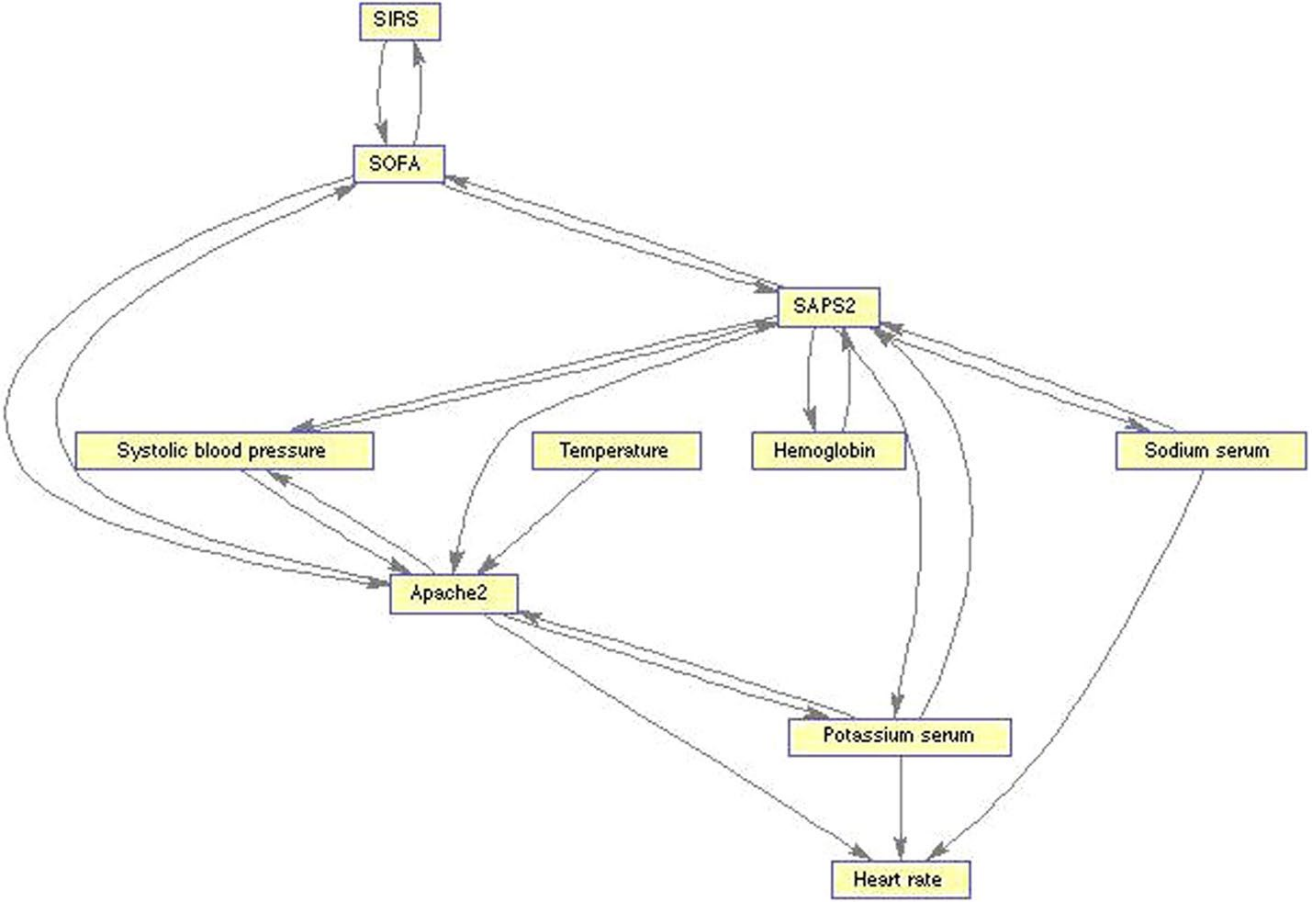
Conditional Independence Map. Conditional Independence Map for organ dysfunction at ICU admission

Analyzing how would SIRS relate to organ dysfunction measured through the SOFA score (Fig. 2 is also of great importance. The resulting TPDA graph here also shows a strong relation between SIRS and SOFA; the statistical dependence of SIRS with the SOFA score is therefore clear. It is also important to note, from this graph, the strong relationship between the SOFA score and severity as measured by the APACHE II [82] and SAPS II [83].

The application of Causal Explorer to the analysis of the MEDAN dataset provides evidence supporting the very recent official modifications of the clinical definition of sepsis [76]. It does so in the sense that SIRS relates to organ dysfunction and, therefore, it is convenient to assign more prominence to the latter for its diagnosis. However, it is also important to highlight the role played by the inflammatory response in the patient outcomes as well as in the physiopathology of sepsis.

#### Finding sepsis prognostic factors using ML methods

This section provides an overview of the use of a latent model-based feature extraction approach, Factor Analysis (FA), to obtain new prognostic factors, understood as sets of descriptors, for the prediction of sepsis-related mortality. Importantly, the results obtained using this approach are readily interpretable for medical experts.

In the experiments reported here [84], the prognostic factors obtained by FA were used to predict mortality using Logistic Regression (LR), a method that is commonly employed in medical applications [85, 86] and widely trusted by clinicians. The prediction results improved on those obtained with current standard data descriptors, which justifies the use of the newly obtained factors as risk-of-death predictors at the ICU.

In this work, a prospective observational cohort study of adult patients with severe sepsis was analyzed. The study was conducted at the Critical Care Department of the Vall d’Hebron University Hospital (Barcelona, Spain), and it was approved by the Hospital Research Ethics Committee. The database includes data from patients with severe sepsis and septic shock, both collected at the ICU by the Research Group in Shock, Organic Dysfunction and Resuscitation (SODIR), between June, 2007 and December, 2010. During this period, 354 patients with severe sepsis (medical and surgical patients) were admitted in the ICU. These data consistently show the worst values during the first 24 hours of evolution for severe sepsis for all variables. Organ dysfunction was evaluated using SOFA scores, while severity was evaluated using the APACHE II score. A total of 34 features was used for the mortality prediction analyses. They are listed in full in [84].

#### ML and interpretability: clinical factor interpretation

The FA outcome was a consistent 14-factor model. The cumulative proportion of total (standardized) sample variance explained by this model was found to be 83.27%.

Considering only the highest factor loadings (in absolute value) for every given variable, these factors were mapped into different readily interpretable clinical descriptors, which can be explained as follows (Note that the original variables with factor loadings bigger than 0.25 are listed in brackets):

- Factor 1: Related to cardiovascular function (*cardiovascular SOFA score* and use of *vasoactive drugs*).
- Factor 2: Corresponds to haematologic function (*haematologic SOFA score* and the *total platelet count*).
- Factor 3: Corresponds to respiratory function (*Respiratory SOFA score*, *mechanical ventilation* and $PaO_2/FiO_2$ *ratio*).
- Factor 4: (*mechanical ventilation* and *PPlateau*).
- Factor 5: Related to the micro-organism producing the sepsis and whether this sepsis is polymicrobial (*germ class* and *polimicrobial infection*).
- Factor 6: (*24 h SSC bundles* and *glycaemic indices*).
- Factor 7: Corresponds to renal function (*renal SOFA score* and *total SOFA score*).
- Factor 8: Related to the hepatic function (*Hepathic SOFA score* and $\hbox {O}_2$ *central venous saturation*).
- Factor 9: Corresponds to the administration of antibiotics and haemocultures taken during the first 6 h of ICU stay (*Antibiotics 6 h*, *haemocultures 6 h*).
- Factor 10: Relates to the number of organs in dysfunction (*Dysfuctioning organs for SOFA 1-2* and *total number of dysfunctioning organs*).
- Factor 11: Corresponds to the CNS function (*Age*, *CNS SOFA score* and *total number of dysfunctioning organs*).
- Factor 12: Related to the loci of sepsis (*Sepsis focus* and *base pathology*).
- Factor 13: (*APACHE II score* and *worst lactate levels*).
- Factor 14: Weak loadings (*Sepsis focus*, *Hepathic SOFA score* and *transfussions 6 h*).

The FA method obtained factors that were coherent with the SOFA score as a description and measure of organ failure and dysfunction [78], combined with the management guidelines defined by the Surviving Sepsis Campaign [87]. It can thus be safely concluded that they are related to SOFA and the actions taken to mitigate this organ deterioration. Such result is particularly interest because among the main challenges in mortality prediction, we find that of producing flexible models that can robustly fit the observed data without requiring any superfluous contextual assumptions, and in the presence of subtle interactions between covariates.

#### Mortality prediction using logistic regression over the 14 extracted factors

Following the FA analysis, mortality prediction using LR was carried out using the 14-factor solution as a starting point. Performance was assessed through 10-fold cross validation. Table 1 shows the coefficient estimates $\beta$, Z-Scores and maximum and minimum values of the LR model fitting to the 14 factors, as well as the ICU outcome (output) and removing those factors yielding Z-Scores smaller than 1.96. These Z-Scores gauge the effect of removing one factor from the model [88, 89] at a time. A Z-score greater than 1.96 in absolute value is significant at the 5% level and provides a measure of the prediction capability of a given factor.

**Table 1:** Results for LR over latent factors with 10-fold cross validation

	$\beta$ coeff	Max	Min	Z-score
Intercept	1.22	1.53	.87	7.11
F4	$-$ 0.54	$-$ 0.23	$-$ 0.86	$-$ 3.38
F10	$-$ 0.69	$-$ 0.38	$-$ 1.05	$-$ 4.26
F9	$-$ 0.51	$-$ 0.21	$-$ 0.81	$-$ 3.36
F13	$-$ 0.49	$-$ 0.24	$-$ 0.74	$-$ 3.80

As shown in Table 1, factor 3, which is related to *Mechanical Ventilation* and *Pplateau*, together with factor 13, which is related to the APACHE II score, showed the strongest effects. Factor 8 (Hepatic Function measured with the SOFA Score) and factor 10 (related to the number of Dysfunctional Organs) were also found to be quite relevant. Note that the factors related to the *Surviving Sepsis Campaign* show no strong effect on mortality prediction using LR. This may be due to the low compliance with the *Surviving Sepsis Campaign Bundles* for the first 6 and 24 hours of evolution (26.18% and 44.06% respectively for the ICU under study). However, it should be pointed out that factor 9 (antibiotic administration and haemocultures) has a higher impact than factor 6 (24 h. bundles with glycaemic indexes). For the ICU under analysis, 80.22% of patients received antibiotics during the first 6 h of evolution and 77.14% had haemocultures during the same period of time. In fact, timely administration of antibiotics and performance of haemocultures are considered critical to the improvement of septic patients’ prognosis. A 10-fold cross validation yielded an Area Under the ROC Curve (AUC) of 0.78. A decision threshold of $\gamma =0.68$ was automatically selected (for the maximization of the discrimination probability) to decide patients’ survival. This 10-fold cross-validation experiment yielded an AUC of 0.78, an error rate of 0.24, a sensitivity of 0.65 and a specificity of 0.80. The results of LR over latent factors can be found in Table 1. This table also shows that the two most representative factors are F10 and F13, which correspond to organ dysfunction measured through the SOFA score and illness severity measured through the APACHE II score combined with the worst lactate levels.

*Comparison with LR over a selection of the original variables*

Experiments continued aiming to compare the predictive ability of the FA 14-factor solution with that which could be achieved using the original data variables. For that, the most relevant clinical features were selected following a backward feature selection process (the backward feature selection removes those variables yielding non-significative Z-scores). According to this, the selected attributes were: the total number of dysfunctional organs; the APACHE II score; and the worst lactate levels. The corresponding coefficients, maximum and minimum values and Z-scores for these three variables can be found in Table 2.

**Table 2:** Results for LR with 10-fold cross validation

	$\beta$ coeff	Max	Min	Z-score
Intercept	4.20	3.11	5.29	7.56
APACHE II	$-$ 0.08	$-$ 0.13	$-$ 0.04	$-$ 3.77
Worts Lact.	$-$ 0.25	$-$ 0.38	$-$ 0.11	$-$ 3.63

LR applied to the most significant attributes a 10-fold cross validation resulted in an AUC of 0.75, which is a worse result than the one obtained from the FA solution. Following the procedure outlined in the previous subsection, a decision threshold of $\gamma =0.68$ was automatically selected. This resulted in a prediction error over the test data of 0.3 (higher than that of the FA solution), a specificity of 0.72, and a sensitivity of 0.64.

*Comparison with the APACHE II mortality score*

The Risk-of-Death (ROD) formula, based on the APACHE II score, is defined as [82]:

1 $$\begin{aligned} \ln \left( \frac{ROD}{1-ROD}\right) =-3.517+0.146\cdot A+\epsilon , \end{aligned}$$

where *A* is the APACHE II score and $\epsilon$ is a correction factor depending on clinical traits at admission in the ICU. For instance, if the patient has undergone post-emergency surgery, $\epsilon$ is set to 0.613. The application of this formula with a threshold of $\gamma =-\,0.25$ to the patients dataset yielded an error rate of 0.28 (higher than the FA solution), a sensitivity of 0.82 and a specificity of 0.55. The corresponding AUC was 0.70.

#### Sepsis mortality prediction from observed data

All these previously reviewed studies investigated the dependence relations between the observed variables and clinical traits and exploited their marginalization to study sepsis and its prognosis through CIM, FA, LR and the APACHE II score. In this section, the use of kernel methods to analyse the same data is explored.

In [67], data were first embedded in a suitable feature space; then algorithms based on linear algebra, geometry and statistics for inference were used. Even from this informal definition, it becomes apparent that all the methods used so far could be kernelized provided the appropriate mappings, spaces, measures and topologies were used. Given the simplicity of the models (only multinomial and multivariate Gaussian distributions are considered, all of which can be efficiently modelled algebraically using the Regular Exponential Family), we proposed to use a generative approach and exploit the inner data structure to build a set of efficient closed-form kernels best suited for these two distributions. More specifically, the performance of the Quotient Basis Kernel (QBK) [67], the simplified Fisher kernel against other state-of-the art methods such as, support vector machines with a Gaussian, Polynomial and linear kernels, generative kernels based on the Jensen-Shannon metric (Centred, Inverse and Exponential kernels) [90] and RVM [63] were all assessed as sepsis mortality predictors.

**Table 3:** Summary of prognosis indicators and their corresponding accuracies

Method	AUC	Error rate	Sens.	Spec.
LR-FA	0.78	0.24	0.65	0.80
LR	0.75	0.30	0.64	0.72
APACHE II	0.70	0.28	0.82	0.55
RVM	0.86	0.18	0.67	0.87
SVM-Quotient	0.89	0.18	0.70	0.86
SVM-Fisher	0.76	0.18	0.68	0.86
SVM-EXP	0.75	0.21	0.70	0.82
SVM-INV	0.62	0.22	0.70	0.82
SVM-CENT	0.75	0.21	0.70	0.82
SVM-GAUSS	0.83	0.24	0.65	0.81
SVM-LIN	0.62	0.26	0.62	0.78
SVM-POLY	0.69	0.28	0.71	0.76

In this case, the data for analysis come from the MIMIC II [91–93] publicly-accessible database, a reference in critical care research. More in particular, the database wa queried for septic patients, obtaining 2,002 entries and 6 predictors (SOFA and SAPS at ICU admission, as well as their corresponding minimum and maximum values during ICU stay). All model performances were evaluated over a random test population consisting of 15% of all the available data. Models were again trained with 10-fold cross-validation and using 70% of data for training and 15% of the data for validation. Table 3 summarizes the results for all experiments.

## Conclusions

The current data availability surplus in medicine in general and critical care in particular poses increasingly complex challenges. ML and related data analysis approaches have provided, over time, a substantial corpus of evidence to support their value in the role usable knowledge extraction from medical data. In this paper, we have surveyed in some detail not only this value, but also the main challenges they face.

The potential of ML in the area has been exemplified by focusing on the sepsis pathology in particular. The new definitions of sepsis and the relevance of using SIRS in its diagnosis have, of late, being a matter of hot debate in the critical care research community. To shed some light on this debate, we have used conditional independence models to analyze sepsis data, showing evidence of a dependence of SIRS on both organ dysfunction measured through the SOFA score and on the ICU outcome. In the light of these results, it is our opinion that the SIRS should still be considered in the study of the pathophysiology of sepsis.

One of the main limitations of the current ICU application of quantitative methods to the assessment of ROD is their lack of specificity (that is, the undesirable high number of false positive cases they churn), which not only puts an extra risk on an already severely affected patient population, but also results in an unnecessary burden for National Health Systems. In this regard, ML and related techniques can play an important role by improving overall performance through a combination of the indicators already in use with further clinical variables, all of which are routinely measured at the point of care. In this paper, we have attempted to show how a representation over latent factors, extracted from the originally mesured features, may be useful. Other possible approaches, very well suited to medical protocols due to their interpretability and explanatory power, are those based on Classification and Regression Trees, as shown in the state-of-the art. Along this lines, it may be also interesting to use Gradient Boosting Trees or Random Forest models for parameter selection and prognosis assessment in future research.

Attending to the nature of clinical data available in the ICU, it might be possible to better assess prognosis through a proper embedding of the data. The techniques presented in the case study reported in this paper are related to regular exponential families in general and to the multinomial and Gaussian exponential families that resulted in the generative kernels outlined in previous sections. The accuracy of routinely used methods for assessing prognosis of septic patients in the ICU (LR and the APACHE II) yield acceptable accuracy, but their performance in terms of specificity is still limited. The RVM and LR models applied over latent factors have been shown to yield an acceptable performance. However, the kernel methods have shown to provide the best balance in all these performance parameters. It is also worth mentioning that the QBK and the simplified Fisher kernel yielded the best results, as reported in Table 3 and that both QBK and the simplified Fisher kernel can be represented through graphical models, increasing their interpretability. Interpretability (sometimes also referred to as explainbility) is a key requirement for complex models that aim to be adopted in actual clinical practice.

In conclusion, we believe that data ML methods have proven to be valuable in an extremely data-intensive environment such as the ICU. They can provide actionable and interpretable prognostic indicators for sepsis and also insight on the relevance of different clinical traits that are not recommended to be used in the current protocols such as the SIRS. In this regard, we believe that future work in the use of ML for the study the role of omics data (from transcriptomics, proteomics and metabolomics) during the inflammatory cascade during sepsis may further improve our understanding of the mechanisms and physiopathology of sepsis.
